# Epithelioid angiosarcoma of the duodenum: a case report

**DOI:** 10.1186/s40792-022-01391-z

**Published:** 2022-02-28

**Authors:** Shinya Sakamoto, Takehiro Okabayashi, Motoyasu Tabuchi, Kenta Sui, Takahiro Murokawa, Jun Iwata

**Affiliations:** 1 Department of Gastroenterological Surgery at Kochi Health Sciences Center, 2125-1 Ike, Kochi-City, Kochi 781-8555 Japan; 2 Department of Diagnostic Pathology at Kochi Health Sciences Center, 2125-1 Ike, Kochi-City, Kochi 781-8555 Japan

**Keywords:** Angiosarcoma, Epithelioid angiosarcoma, Duodenal tumor

## Abstract

**Background:**

Angiosarcomas are rare malignant tumors that arise from the endothelium of blood vessels. They occur most commonly in the skin and soft tissue, and less commonly in the breast, liver, bone, and spleen. Gastrointestinal angiosarcomas are extremely rare. Herein, we present a case of duodenal epithelioid angiosarcoma that was treated with surgical resection.

**Case presentation:**

A 68-year-old man presented with a 1-month history of fatigue and hypotension. He visited the outpatient clinic for a routine follow-up. Laboratory examination revealed anemia. Esophagogastroduodenoscopy revealed multiple duodenal lesions with central ulceration. A biopsy showed a sheet-like arrangement of large round and spindle-shaped tumor cells that were positive for CD31. Based on the histological and immunohistochemical staining findings, an epithelioid angiosarcoma was diagnosed. Computed tomography (CT) and positron emission tomography–CT revealed no lymph node metastasis or distant metastasis. Radical subtotal stomach-preserving pancreatoduodenectomy with lymphadenectomy was performed. After removing the specimen, reconstruction was performed using the Child procedure. Grossly, two dark-red polypoid tumors were found in the second portion of the duodenum. Histological evaluation revealed proliferation of malignant round and polygonal cells arranged in sheets and spindle-like cells arranged in bundles. Vasoformative structures were recognized as slit-like spaces containing red blood cells. Immunohistochemical staining demonstrated that the tumor cells were positive for CD31. These findings confirmed the diagnosis of epithelioid angiosarcoma in the duodenum. The patient’s postoperative course was uneventful. The patient was discharged on postoperative day 19 without any complications. At a follow-up examination in the outpatient clinic at postoperative 4 months, no evidence of recurrence was detected.

**Conclusion:**

The present report describes a case of duodenal epithelioid angiosarcoma. Duodenal angiosarcomas may cause anemia and gastrointestinal bleeding. Because angiosarcomas sometimes show epithelioid cytomorphology, immunohistochemical analysis is useful for confirming the diagnosis.

## Background

Angiosarcomas are rare malignant tumors that arise from the endothelium of blood vessels. These tumors account for 1–2% of all soft tissue sarcomas [1]. They occur most commonly in the skin and soft tissue, and less commonly in the breast, liver, bone, and spleen [2]. Gastrointestinal angiosarcomas are extremely rare. Herein, we present a case of duodenal epithelioid angiosarcoma that was treated with surgical resection. We have described the pathological features of epithelioid angiosarcoma and its clinical features, including endoscopic findings.

## Case presentation

**Fig. 1:**
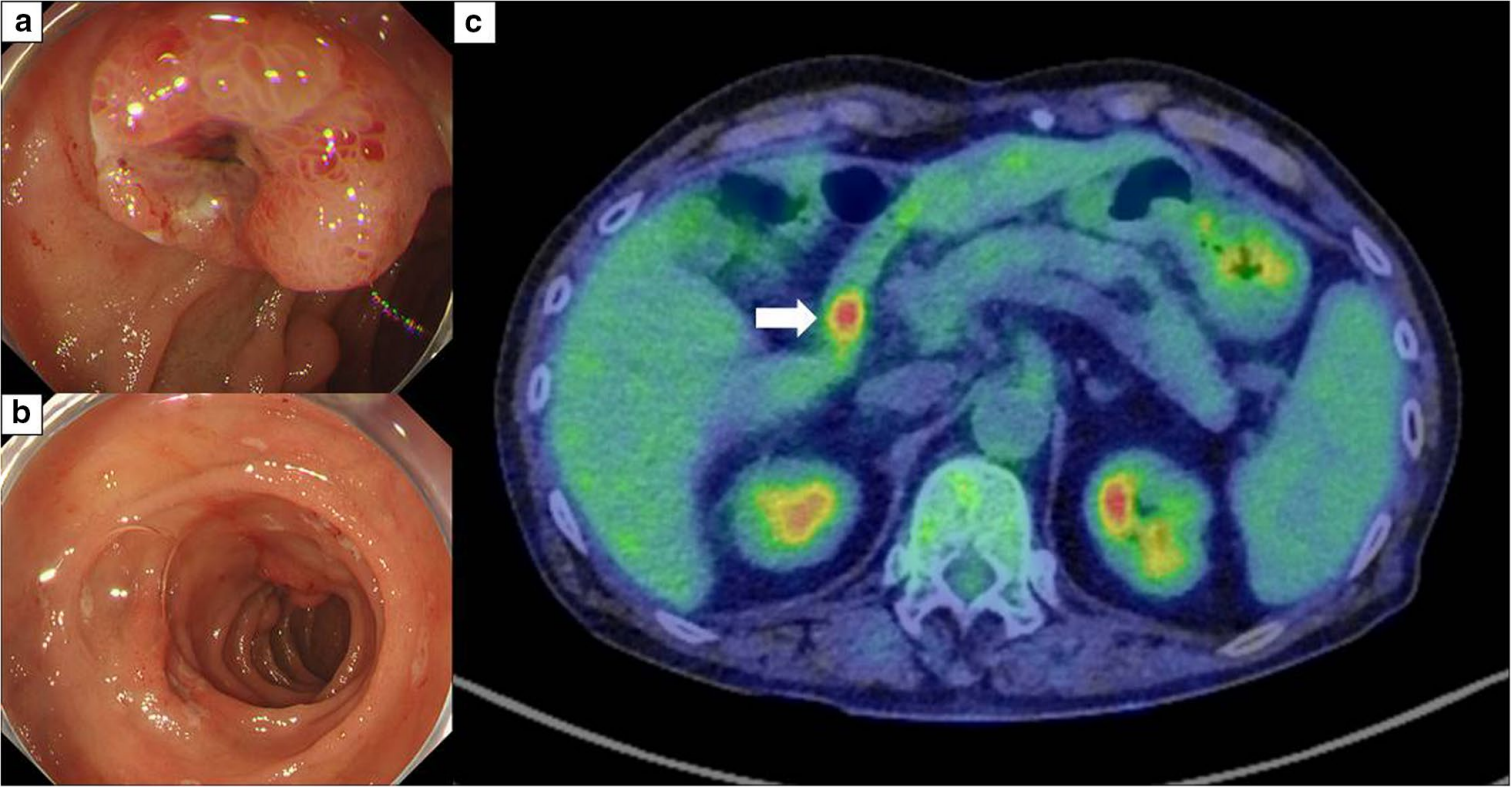
Preoperative examination. **a**, **b** Esophagogastroduodenoscopy (EDGS) images: EDGS detected two reddish elevated lesions with central ulceration in the duodenum. **a** The anal side tumor was reddish elevated lesions with central ulceration. The oral side tumor was ulcerative lesion with slightly elevated red border. **c** Positron emission tomography-CT (PET-CT) image: PET-CT showed increased uptake (mean maximum standardized uptake value [SUV_max_]: 6.25) of the oral side tumor in the duodenum

**Fig. 2:**
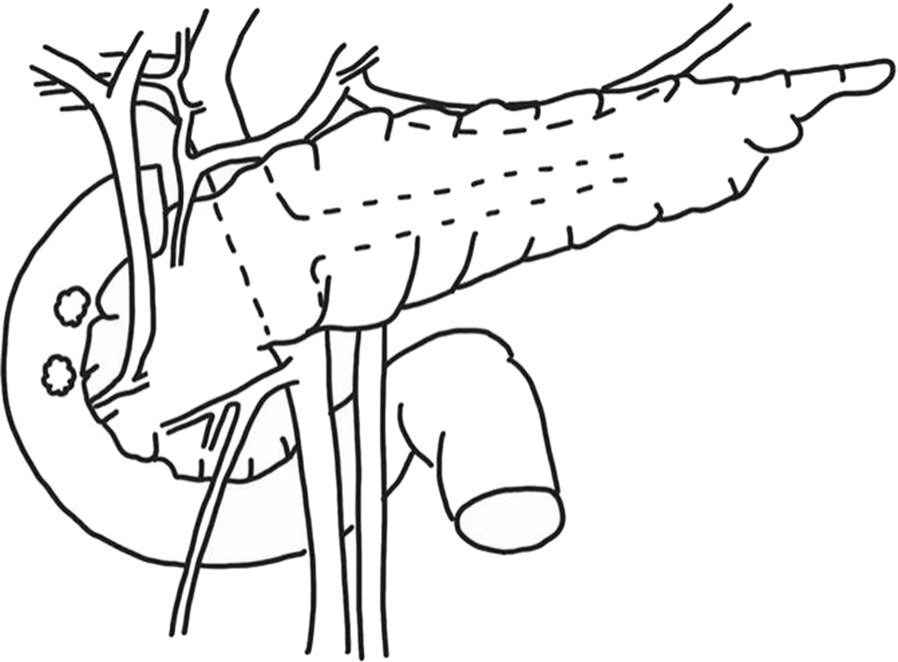
Schema demonstrates localization of two duodenal lesions

A 68-year-old man presented with a 1-month history of fatigue and hypotension. He underwent aortic root replacement for annuloaortic ectasia 9 years prior and underwent omentum patch repair for sternal osteomyelitis after sternotomy. He had a medical history of essential hypertension and pneumoconiosis. He took aspirin, warfarin, some hypertensive drugs, and proton pump inhibiter. He visited the outpatient clinic for a routine follow-up. Laboratory investigation showed a hemoglobin level of 8.7 g/dL, a significant decrease compared with the previous value of 15.2 g/dL. After he was documented anemia, he stopped to take warfarin and continued to take aspirin for antithrombotic drugs due to cardiac surgeon’s decision. Esophagogastroduodenoscopy (EDGS) revealed multiple duodenal lesions with raised red borders and central depressed areas. The anal side tumor was elevated lesions with central depressed area (Fig. 1a). The oral side tumor was ulcerative lesion with slightly elevated red border (Fig. 1b). Both tumors located in the second portion of the duodenum (Fig. 2). An abdominal computed tomography (CT) scan showed no intraluminal mass or wall thickening in the duodenum. Positron emission tomography–CT (PET–CT) demonstrated increased uptake in the duodenum (Fig. 1c). No evidence of lymph node metastasis or distant metastasis was observed. A biopsy showed a sheet-like arrangement of large round and spindle-shaped tumor cells, and the tumor cells were positive for CD31. Based on the histological and immunohistochemical staining findings, an epithelioid angiosarcoma was diagnosed. Surgical resection was performed.

**Fig. 3:**
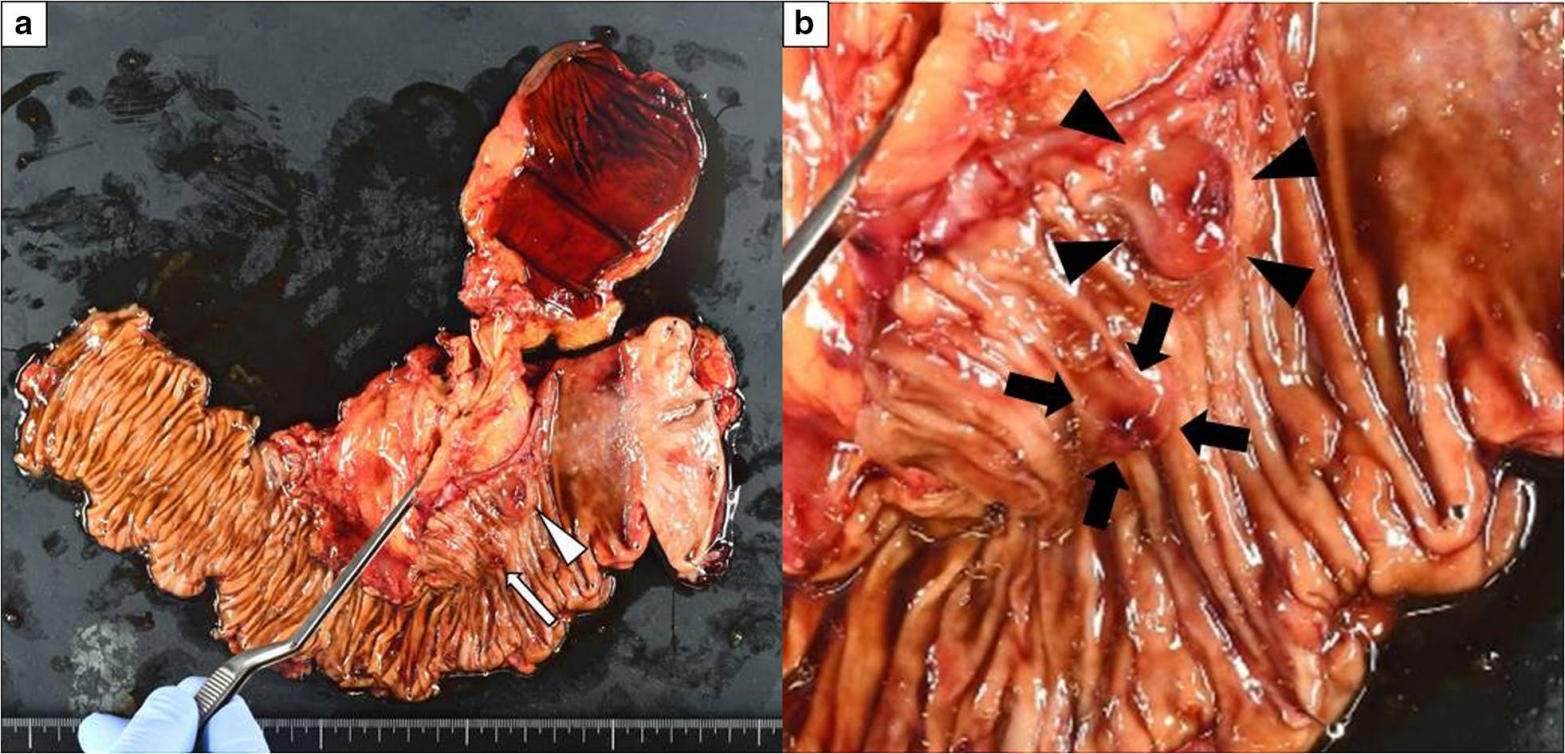
Resected specimen. **a** Surgical specimen from subtotal stomach-preserving pancreatoduodenectomy. Two dark red polypoid tumors were found in the duodenum. Arrowhead; the oral side tumor. Arrow; the anal side tumor. **b** The anal side tumor (arrow) showed erosion, and the oral side tumor (arrowhead) showed ulceration

Exploratory abdominal surgery revealed no peritoneal metastases. The omentum, which was patched to the sternum, was adherent to the liver. Adhesiolysis of the upper abdominal cavity was initially performed, followed by radical subtotal stomach-preserving pancreatoduodenectomy with lymphadenectomy. We dissected *no. 5, 6, 8a, 8p, 12a, 12p, 12b, 13a, 14p, 17a* lymph nodes [3]. After removing the specimen, reconstruction was performed using the Child procedure (Fig. 3a).

**Fig. 4:**
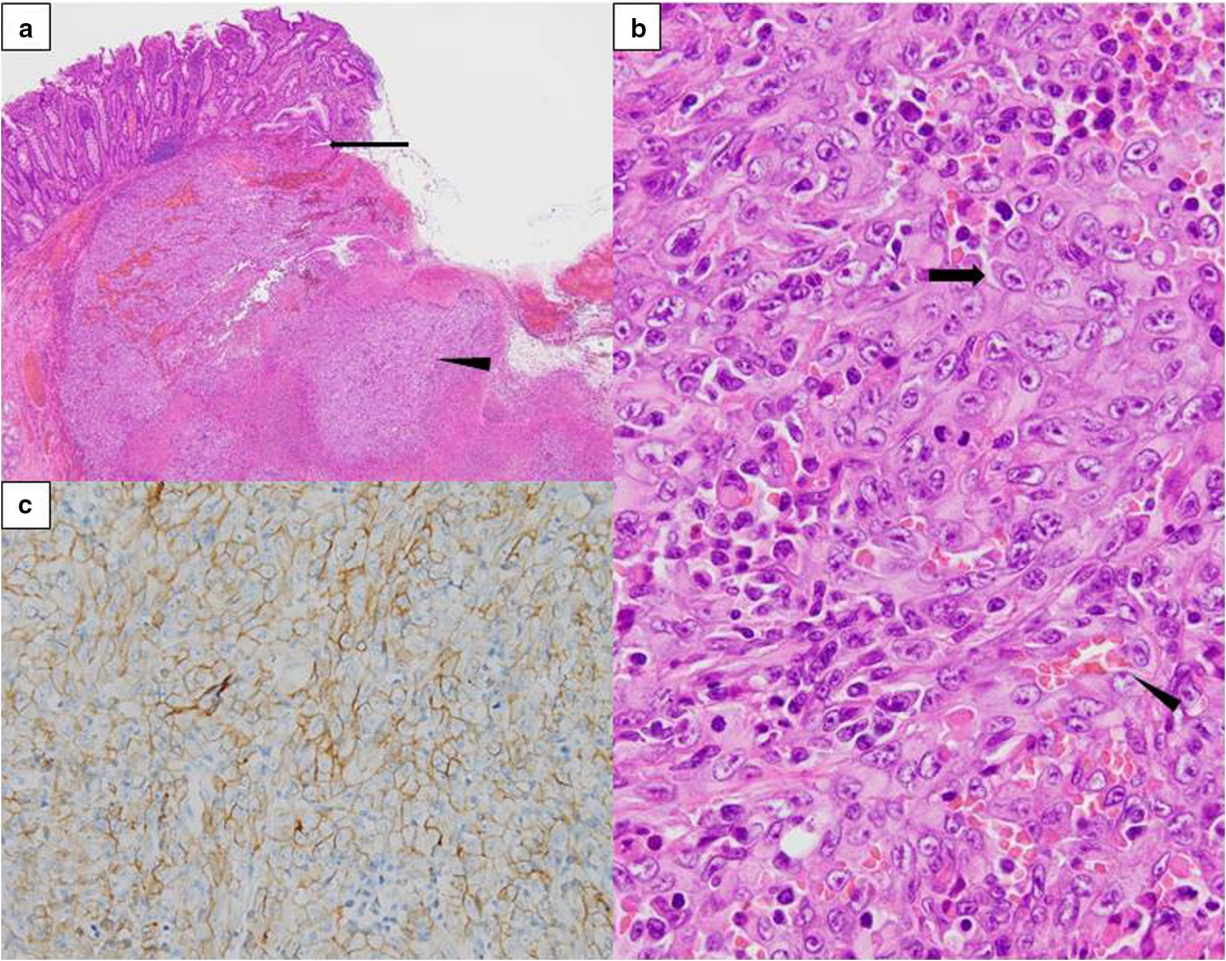
Histological findings. **a** Microscopic image of the oral side tumor revealed that the tumor primarily arise from submucosal layer (hematoxylin–eosin [H&E] stain; 4×). Arrow; muscularis mucosa. Arrowhead; tumor cells. **b** Higher power image of the oral side tumor revealed large round and polygonal cells with moderate amounts of cytoplasm arranged in sheets (arrow), and a slit-like space contained red blood cells (arrowhead) (H&E stain; 40×) **c** Tumor cells were immunoreactive for CD31

Grossly, two dark-red polypoid tumors were found in the second portion of the duodenum. The gross appearance was slightly different from EGDS findings. The anal side tumor, measuring 1.0 × 1.0 cm, showed erosion, and the oral side tumor, measuring 1.4 × 0.9 cm, was ulceration (Fig. 3b). The lesions appeared to be centered in the submucosal layer, with expansion up to the mucosa (Fig. 4a). No spatial continuity was found between the two tumors. Histological evaluation revealed proliferation of malignant round and polygonal cells arranged in sheets and spindle-like cells arranged in bundles. Vasoformative structures were recognized as slit-like spaces containing red blood cells (Fig. 4b). No lymphatic or venous invasions were detected in both tumors. Immunohistochemical staining demonstrated that the tumor cells were positive for CD31, CK (AE1/AE3), and CAM5.2, and negative for CD34 and EMA (Fig. 4c). The lymph nodes revealed no tumor metastasis. These findings confirmed the diagnosis of epithelioid angiosarcoma in the duodenum without lymph node metastasis. The patient’s postoperative course was uneventful. The patient was discharged on postoperative day 19 without any complications. At a follow-up examination in the outpatient clinic at postoperative 4 months, no evidence of recurrence was detected.

## Discussion

Angiosarcoma is a rare form of malignant connective tissue neoplasm that arises from the endothelial cells of blood vessels. Tumors can arise in any part of the body, and many cases are seen on the skin of the head and neck region. Intra-abdominal angiosarcomas are rare, and the spleen and liver are the most commonly associated organs. The gastrointestinal tract is an extremely rare site for angiosarcoma. To the best of our knowledge, only five cases of primary duodenal angiosarcoma have been reported so far [4–8]. In the present case, we performed pancreatoduodenectomy for epithelioid angiosarcoma of the duodenum.

Clinically, angiosarcomas can present with various symptoms depending on their primary location. In most cases of gastrointestinal angiosarcoma, the symptoms are nonspecific, including gastrointestinal bleeding, abdominal pain, intestinal obstruction, abdominal distention, weight loss, shortness of breath, anemia, and weakness [9]. Although gastrointestinal bleeding is the most common chief complaint due to the intraluminal invasion of the tumor, cases of intraperitoneal bleeding caused by gastrointestinal angiosarcoma have also been reported [10, 11]. Gastrointestinal angiosarcomas appear as centrally ulcerated nodules, hemorrhagic nodules, or highly erythematous or purpuric nodules [12]. In the current case, the patient presented with anemia due to gastrointestinal bleeding. EGDS revealed a reddish polypoid lesion with a central ulcer, which was similar to a previous report [5, 6].

The histologic features of angiosarcoma show a wide spectrum of appearances, ranging from clear vasoformative to poorly differentiated solid tumors in which the vascular nature is not readily apparent. Some angiosarcomas demonstrate epithelioid cytomorphology. Epithelioid angiosarcoma is composed of solid sheets of large, oval, or rounded epithelioid cells with abundant eosinophilic or amphophilic cytoplasm and a large, pale vesicular nucleus with a conspicuous eosinophilic nucleolus [13]. However, because of the variegated histological features, diagnosis may be difficult to achieve based on pure morphology. Immunohistochemical analysis is useful for confirming the diagnosis of epithelioid angiosarcoma. CD31, ERG, vimentin, factor VIII-related antigen, and Ulex europaeus agglutinin-1 (UEA-1) are sensitive and reliable markers for the immunohistochemical evaluation of epithelioid angiosarcoma [14–16]. In the current case, immunohistochemical workup was carried out in the initial small biopsy specimen; thus, we could diagnose this tumor as epithelioid angiosarcoma pre-operatively. Vasoformative structures and solid growth pattern of spindle-shaped cells and large polygonal epithelioid-type cells were seen in the tumors from the resected specimen. Immunohistochemical staining demonstrated that the tumor cells were positive for CD31. Therefore, a duodenal epithelioid angiosarcoma was diagnosed. Angiosarcoma should be kept in mind, especially when clues to angioformative morphology are noted, and a well-directed immunohistochemical workup is required even in a biopsy specimen.

Angiosarcomas are known for their rapid proliferation, aggressive infiltration, hematogenous metastasis, and relatively poor prognosis with low life expectancy. The 5-year survival rate is 31–40% with a median overall survival of 16–31 months [17, 18]. The primary treatment modality for angiosarcoma is surgical excision with clean margin [2]. However, even for local disease, the survey suggests that only 60% of patients survive for more than 5 years [19]. For gastrointestinal angiosarcoma cases, complete surgical resection is considered the only factor that correlates with disease-free survival [20]. Although the effectiveness of lymphadenectomy for gastrointestinal angiosarcoma remains unclear, regional lymph nodes were reported one of the most common recurrence sites for angiosarcomas of bone or soft tissue [21–23]. Therefore, gastrointestinal angiosarcoma might have potential to metastases to regional lymph node. In the current case, we performed pancreatoduodenectomy with lymphadenectomy and achieved complete surgical resection. The patient was discharged without any complications, and the postoperative course was generally favorable.

In conclusion, the present report describes a case of duodenal epithelioid angiosarcoma. Duodenal angiosarcomas may cause anemia and gastrointestinal bleeding. Because angiosarcomas sometimes show epithelioid cytomorphology, immunohistochemical analysis is useful for confirming the diagnosis.

## Data Availability

All data generated or analyzed during this study are included in the published article.

## Abbreviations

EGDS: Esophagogastroduodenoscopy
CT: Computed tomography
PET-CT: Positron emission tomography- computed tomography
UEA-1: Ulex europaeus agglutinin-1
